# Retention on ART and predictors of disengagement from care in several alternative community‐centred ART refill models in rural Swaziland

**DOI:** 10.1002/jia2.25183

**Published:** 2018-09-18

**Authors:** Lorraine Pasipamire, Robin C Nesbitt, Sindiso Ndlovu, Gibson Sibanda, Sipho Mamba, Nomthandazo Lukhele, Munyaradzi Pasipamire, Serge M Kabore, Barbarba Rusch, Iza Ciglenecki, Bernhard Kerschberger

**Affiliations:** ^1^ Médecins Sans Frontières Mbabane Swaziland; ^2^ Médecins Sans Frontières Nhlangano Swaziland; ^3^ Swaziland National Aids Program (SNAP) Ministry of Health Mbabane Swaziland; ^4^ Médecins Sans Frontières Geneva Switzerland

**Keywords:** HIV care continuum, community, retention, differentiated care, Swaziland, models of community ART delivery

## Abstract

**Introduction:**

A broad range of community‐centred care models for patients stable on anti‐retroviral therapy (ART) have been proposed by the World Health Organization to better respond to patient needs and alleviate pressure on health systems caused by rapidly growing patient numbers. Where available, often a single alternative care model is offered in addition to routine clinical care. We operationalized several community‐centred ART delivery care models in one public sector setting. Here, we compare retention in care and on ART and identify predictors of disengagement with care.

**Methods:**

Patients on ART were enrolled into three community‐centred ART delivery care models in the rural Shiselweni region (Swaziland), from 02/2015 to 09/2016: Community ART Groups (CAGs), comprehensive outreach care and treatment clubs. We used Kaplan–Meier estimates to describe crude retention in care model and retention on ART (including patients who returned to clinical care). Multivariate Cox proportional hazard models were used to determine factors associated with all‐cause attrition from care model and disengagement with ART.

**Results:**

A total of 918 patients were enrolled. CAGs had the most participants with 531 (57.8%). Median age was 44.7 years (IQR 36.3 to 54.4), 71.8% of patients were female, and 62.6% fulfilled eligibility criteria for community ART. The 12‐month retention in ART was 93.7% overall; it was similar between model types (*p* = 0.52). A considerable proportion of patients returned from community ART to clinical care, resulting in lower 12 months retention in care model (82.2% overall); retention in care model was lowest in CAGs at 70.4%, compared with 86.3% in outreach and 90.4% in treatment clubs (*p* < 0.001). In multivariate Cox regression models, patients in CAGs had a higher risk of disengaging from care model (aHR 3.15, 95% CI 2.01 to 4.95, *p* < 0.001) compared with treatment clubs. We found, however, no difference in attrition in ART between alternative model types.

**Conclusions:**

Concurrent implementation of three alternative community‐centred ART models in the same region was feasible. Although a considerable proportion of patients returned back to clinical care, overall ART retention was high and should encourage programme managers to offer community‐centred care models adapted to their specific setting.

## 1 Introduction

Swaziland, the country with the highest HIV prevalence in the world (30.5% in adults aged 18 to 49 years) 1, adopted the World Health Organization (WHO) “treat‐all” strategy 2 in October 2016. This treatment approach provides antiretroviral therapy (ART) to all people living with HIV (PLHIV) at the time of HIV diagnosis regardless of CD4 cell count and WHO staging criteria 2. The WHO also advocates for differentiated care which aims to provide a more patient‐centred approach to HIV care and ART delivery 2. Differentiated care includes a range of community‐centred ART delivery models for patients stable on ART, often defined as patients who have received ART for at least one year without adverse drug reactions, have a good understanding of lifelong adherence and evidence of treatment success 2. The care models may differ in content, location, provider and frequency of services delivered but all share the aim of decreasing ART refill visits for patients and decreasing patient load for healthcare workers 2, 3, 4, 5.

Implementation of patient‐centred ART care has been reported from several settings in sub‐Saharan Africa 6, 7, 8, 9, 10, 11, 12, 13, 14, 15, 16, 17, 18, 19. Community ART Groups (CAGs) have been piloted in Malawi, Lesotho and Mozambique as a way to reduce patient time and costs spent travelling and queuing for ART refills 7, 10, 11, 18. In Mozambique the CAG model retained 97.7% and 91.8% of patients at 12, and 48 months 12. Facility‐based treatment clubs, also known as adherence clubs, were piloted in South Africa and Kenya with the aim to decongest facilities, increase retention in care and lower service provider cost 13, 14, 15, 19, 20. The results showed 97% retention in care for the club patients compared with 85% for other patients not enrolled in the model 13. Other alternative models have been reported elsewhere including fixed community points, mobile outreach ART delivery and home delivery 8, 16, 17, 21, 22.

The introduction of more patient‐centred care models, however, bring challenges to already over‐burdened health systems in low‐ and middle‐income countries. There is little reported experience on the feasibility of providing a combination of community‐centred ART care models in the same setting. This report describes the introduction of several community‐ and facility‐based ART refill models under routine programmatic conditions in the rural Shiselweni region in Swaziland. The objectives were to compare retention in care model and retention on ART among the care models and to determine factors associated with all‐cause attrition.

## 2 Methods

### 2.1 Setting

Since 2008, the Swaziland Ministry of Health and Médecins Sans Frontières decentralized HIV and TB care in the predominantly rural Shiselweni region (population of approximately 204,000)^23^. Provision of ART care was exclusively facility‐based at primary and secondary health facilities with an estimated 24,000 active on ART by the end of 2015 24. In 2015, we introduced community‐centred ART refill models in a routine public health setting in Shiselweni region.

We conducted a retrospective analysis of adults aged 16 years and above enrolled in three different community‐centred ART refill models in the Shiselweni region from February 2015 to August 2016. First, CAGs linked to primary care clinics (n = 16) comprised a maximum of six patients who alternated attending the primary health clinic for consultation and pick up of drugs for the other group members, thus a patient visiting the clinic for consultation twice in a year. Second, comprehensive outreach care, delivered by one primary and one secondary care facility, integrated drug refills into existing mobile clinic outreach providing antenatal, child welfare and HIV testing services to remote communities. Third, facility‐based treatment clubs comprised 30 patients who met every three months at a health facility for one hour for patient education and drug refills. This model was offered at a large health centre with approximately 4000 people on ART. Eligibility criteria for enrolment into the care models were: age 16 years and above, weight above 45 kg, CD4 over 350 cells/μL, on ART for a minimum of 12 months and virologically suppressed. Enrolled patients could return back to routine clinical care either for personal reasons or for clinical reasons namely elevated viral load >1000 copies/mL, occurrence of TB disease or any other opportunistic infections. However, patients enrolled into care models who were found to be ineligible at analysis stage were included in the analysis.

### 2.2 Definitions and statistical methods

For all analyses, follow up began on the date of the first ART model visit and ended with the first of database closure (30/11/2016) or the last visit in case of transfer out, death or lost to follow‐up (LTFU). Patient LTFU was defined as patients without recorded visit for 120 days or more before database closure. In primary analysis (retention in care model), the outcome of interest was time to the composite endpoint of LTFU, death and exit from the specific care model at enrolment. In secondary analysis (retention in ART care), the outcome was time from enrolment to the composite endpoint of LTFU and death, regardless whether the outcome occurred while enrolled in the care model or in routine facility‐based ART care. For both analyses, censoring occurred in the case of transfer out or database closure. For CAGs, a visit from one member of the group was counted as a visit for all members. One day of follow‐up was added for individuals with an outcome (LTFU or death) on the day of first visit in order not to exclude them from survival analysis.

Baseline demographic and treatment characteristics were described using median and interquartile ranges for continuous variables and, frequencies and proportions for categorical variables. Tests for differences in characteristics between models were performed using Kruskal–Wallis tests for continuous variables and Chi squared tests for categorical variables. Retention in community‐centred ART refill care model and retention in ART care were assessed graphically using Kaplan–Meier curves. Cox proportional hazard models were used to determine socio‐demographic and clinical factors associated with attrition. Multivariable models included individual factors, *a priori* including gender, age and clinical variables (CD4 at enrolment, time on ART before enrolment into community ART model, ART regimen, and whether or not the routine eligibility criteria for inclusion in community ART was met). Proportional hazards assumptions were tested for all models. Analyses were done using Stata version 14.1 (Stata‐Corp, College Station, TX, USA).

### 2.3 Ethics

This retrospective analysis of routine data fulfilled the requirements by the Swaziland Ministry of Health National Research Review Board***.*** In addition, this research fulfilled the exemption criteria set by the Médecins Sans Frontières Ethics Review Board (ERB) for *a posteriori* analyses of routinely collected clinical data and thus did not require MSF ERB review.

## 3 Results

**Figure 1:**
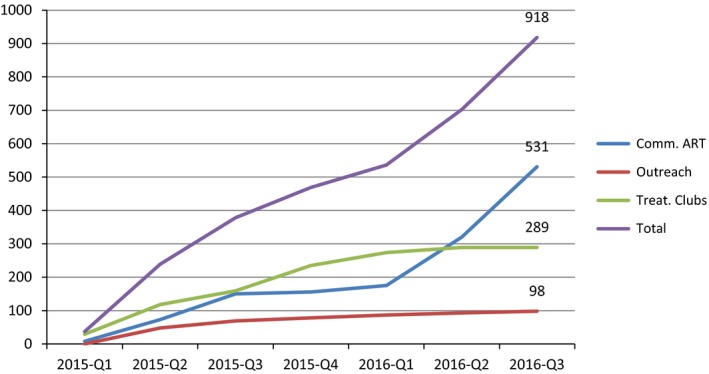
Cumulative enrolment in alternative community‐centred ART models and overall.

A total of 918 patients were enrolled into three community‐centred ART models: 531 (57.8%) in CAGs, 289 (31.5%) in treatment clubs and 98 (10.7%) in comprehensive outreach (Figure 1). The overall median age was 44.7 years (IQR 36.3 to 54.4); participants in treatment clubs were slightly younger and those in CAGs were slightly older (*p* < 0.001, Table 1). Over 70% of patients were female, with no difference in proportion by care model (*p* = 0.06). The overall median CD4 count at enrolment was 477 (IQR 351 to 638) cells/μL, lowest for patients in CAGs (445 cells/μL) and highest for treatment clubs (530 cells/μL, *p* < 0.001). Patient ART regimens also differed between community ART models (Table 1). Overall participants had been on ART for a median of 5.6 years (IQR 3.6 to 7.3) before joining one of the alternative care models, with no difference in time on ART between models (*p* = 0.12). Eligibility criteria for enrolment were met for 575 (62.6%) patients. The most common eligibility criterion not met in 343 patients (78.4%) was CD4 count more than 350 cells/μL, 74 patients had weight less than 45 kg (21.6%), 28 patients were on ART for less than one year or unknown time (8.2%), and 14 patients were less than 16 years (4.1%). Protocol enrolment violations were most common in CAGs, where only 55.9% of patients fulfilled the eligibility criteria (*p* < 0.001, Table 1). Group size differed with model type. CAGs had a median of five members per group (range 3 to 6), treatment clubs had a median of 32 members (range 29 to 35) and comprehensive outreach was available on an individual level at two sites with 29 patients and 69 patients, respectively.

**Table 1:** Baseline characteristics of patients enrolled in alternative ART, by care model

Individual characteristics	CAGs		Comprehensive outreach		Treatment clubs		Total		*p*‐value
	n	%	n	%	n	%	n	%	
Gender									
Male	157	29.6	34	34.7	68	23.5	259	28.2	0.06
Female	374	70.4	64	65.3	221	76.5	659	71.8	
Age group									
≤24 years	22	4.1	4	4.1	10	3.5	36	3.9	0.001
25 to 49 years	292	55.0	60	61.2	204	70.6	556	60.6	
≥50 years	217	40.9	34	34.7	75	26.0	326	35.5	
Median (IQR)	46.5 (38 to 56.6)		44.4 (35.7 to 54.5)		39.9 (34 to 50.4)		44.7 (36.3 to 54.4)		<0.001
CD4 Category (cells/μL)									
<350	157	31.7	20	21.5	39	14.1	216	25.0	<0.001
350 to 500	141	28.5	33	35.5	84	30.3	258	29.8	
>500	197	39.8	40	43	154	55.6	391	45.2	
Median (IQR)	445 (312 to 609)		475 (367 to 639)		530 (418 to 704)		477 (351 to 638)		<0.001
Time on ART before enrolment									
<3 years	98	19.0	26	28.0	46	16.0	170	19.0	0.08
3 to 6 years	181	35.1	30	32.3	122	42.5	333	37.2	
6 to 9 years	178	34.6	26	28.0	83	28.9	287	32.1	
>9 years	58	11.3	11	11.8	36	12.5	105	11.7	
Median (IQR)	5.8 (3.7 to 7.3)		5.2 (2.7 to 6.6)		5.3 (3.6 to 7.3)		5.6 (3.6 to 7.3)		0.12
Inclusion criteria met									
Yes	297	55.9	59	60.2	219	75.8	575	62.6	<0.001
No	234	44.1	39	39.8	70	24.2	343	37.4	
NRTI									
ABC	9	1.7	2	2.1	8	2.8	480	52.3	0.002
AZT	261	49.2	53	54.6	104	36.0	19	2.1	
TDF	261	49.2	42	43.3	177	61.2	418	45.6	
NNRTI/PI									
EFV	333	62.8	58	59.8	156	54.0	547	59.7	0.03
LPVr	6	1.1	4	4.1	5	1.7	15	1.6	
NVP	191	36.0	35	36.1	128	44.3	354	38.7	
Outcomes									
Died	3	0.6	2	2.0	1	0.4	6	0.7	0.001
LTFU	9	1.7	6	6.1	12	4.2	27	2.9	
Transfer out	3	0.6	2	2.0	1	0.4	6	0.7	
Return to clinical care	72	13.6	7	7.1	17	5.9	96	10.5	
Retained in care model	444	83.6	81	82.6	258	89.3	783	85.3	

Missing values: CD4 n = 53, time on ART n = 23, NRTI n = 1, NNRTI==2.

CAGs, community ART groups; LTFU, lost to follow‐up; NRTI, nucleoside reverse transcriptase inhibitor; NNRTI, non‐nucleoside reverse transcriptase inhibitors; PI, protease inhibitors.

### 3.1 Retention in care model

**Figure 2:**
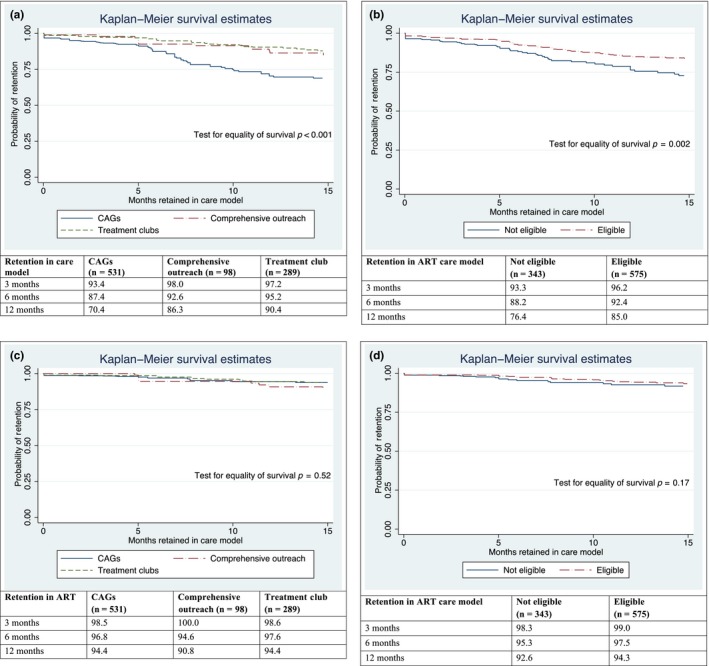
Kaplan–Meier survival curve for retention in community ART care model by (**a**) model type and **(b)** eligibility status. Kaplan–Meier survival curve for retention in ART by (**c**) model type and **(d)** eligibility status.

The overall care model retention was 90.9% and 82.2% at 6 and 12 months, and retention in care model differed significantly by model type, being lowest in CAGs at all time points (*p* < 0.001, Figure 2a). Only 70.4% of patients were retained in CAGs at 12 months compared with 86.3% in comprehensive outreach and 90.4% in treatment clubs. Retention in care model was significantly higher in eligible patients compared with non‐eligible patients (85.0% and 76.4% at 12 months, *p* = 0.017, Figure 2b).

In unadjusted survival models including all patients, care model type and age less than 24 years were associated with disengagement (death, LTFU, return to clinical care) (Table 2). CAGs had more than three times higher hazard rate (HR 3.24, 95% CI 2.11 to 4.97, *p* < 0.001) than treatment clubs and there was no difference between treatment clubs and comprehensive outreach. Non‐eligible patients enrolled in community‐centred ART models had a 71% increase in hazard rate of all‐cause failure (death, LTFU, return to clinical care) compared with those fulfilling eligibility requirements (HR 1.71, 95% CI 1.21,2.42, *p* = 0.003). The effect of model type on retention was slightly attenuated in adjusted models with patients enrolled in CAGs 3.15 times (aHR 3.15, 95% CI 2.01 to 4.95, *p* < 0.001) more likely to disengage (death, LTFU, return to clinical care) than patients in treatment clubs. The effect of eligibility was no longer significant (1.37, 95% CI 0.94 to 2.01, *p* = 0.10). In models of eligible patients only, patients in CAGs were more likely to disengage (death, LTFU, return to clinical care) compared with patients in treatment clubs (aHR 4.29 95% CI 2.38 to 7.71, *p* < 0.001) and no other factors studied were predictive of attrition (Table S1).

**Table 2:** Adjusted and unadjusted Cox model estimates of all‐cause attrition (death, LTFU, disengagement from care model) in care model

	Unadjusted estimates			Adjusted model n = 893		
	HR	95% CI	*p*	HR	95% CI	*p*
Model						
CAGs	3.24	2.11 to 4.96	<0.01	3.15	2.01 to 4.95	<0.001
Outreach	1.52	0.82 to 2.83	0.19	1.39	0.72 to 2.68	0.33
Treat. Clubs	1.0			1.0		
Gender						
Male	1			1		
Female	0.89	0.61, 1.30	0.55	1.03	0.69, 1.53	0.88
Age group (years)						
<24	2.33	1.27, 4.27	0.01	1.73	0.90, 3.34	0.10
25 to 49	1			1		
50+	0.86	0.58, 1.27	0.44	0.76	0.51, 1.14	0.19
CD4 at enrolment						
<350	1					
350 to 500	0.74	0.46, 1.18	0.20			
>500	0.71	0.47, 1.10	0.12			
Time on ART						
0 to 3	1			1		
3 to 6	0.87	0.55, 1.37	0.54	1.07	0.66, 1.72	0.78
6 to 9	0.83	0.51, 1.35	0.46	1.03	0.59, 1.80	0.92
9+	0.45	0.21, 0.99	0.047	0.65	0.29, 1.48	0.31
Eligible						
Eligible	1			1		
Not eligible	1.71	1.21, 2.42	<0.01	1.37	0.94, 2.01	0.10
NRTI						
TDF	1			1		
ABC	1.51	0.61, 3.73	0.38	1.82	0.72, 4.61	0.20
AZT	0.84	0.59, 1.21	0.35	0.90	0.54, 1.51	0.70
NNRTI/PI						
EFV	1			1		
LPVr	1.38	0.44, 4.36	0.59	1.88	0.57, 6.20	0.30
NPV	0.75	0.52, 1.09	0.13	0.90	0.55, 1.49	0.69

The global proportional hazard test for the adjusted model was *p* = 0.25. NRTI, nucleoside reverse transcriptase inhibitor; NNRTI, non‐nucleoside reverse transcriptase inhibitors; PI, protease inhibitor.

### 3.2 Retention in ART

The overall ART retention was 96.7% and 93.7% at 6 and 12 months. It was over 90% for all three models at all time points and there was no difference between care models (*p* = 0.52) (Figure 2c). Retention in ART at 12 months was not statistically different between eligible and non‐eligible patients, with retention at 92.6% and 96.4% respectively (*p* = 0.17, Figure 2d).

In both adjusted and unadjusted models including both eligible and ineligible patients, there was no difference in ART attrition between the care model types (Table 3). In the adjusted model, ART regimens including ABC increased the rate of disengaging with care (aHR 3.91, 95% CI 1.11, 13.75, *p* = 0.034). Older patients were less likely to disengage with care than middle‐aged adults (aHR 0.41 95% CI 0.18, 0.95, *p* = 0.04). Models including eligible patients only showed similar effects, except that the effect of regimen type was attenuated (Table S2). Reasons for returning to clinical care were unknown for 31% of patients (Table 4). Considering known reasons, the most common explanations for returning to clinical care were personal reasons (20.8%), followed by dissolution of the group (19.8%, Table 4).

**Table 3:** Unadjusted and adjusted Cox model estimates of all‐cause ART attrition (death, LTFU)

	Unadjusted estimates			Adjusted model n = 841		
	HR	95% CI	*p*	HR	95% CI	*p*
Model						
CAGs	1			1		
Outreach	1.47	0.66, 3.28	0.35	1.71	0.74, 3.92	0.21
Treat. Clubs	0.93	0.48, 1.82	0.84	0.91	0.45, 1.84	0.80
Gender						
Male	1			1		
Female	0.81	0.44, 1.51	0.51	0.78	0.40, 1.53	0.47
Age‐group years						
<24	2.15	0.84, 5.50	0.11	2.54	0.94, 6.87	0.07
25 to 49	1			1		
50+	0.39	0.17, 0.88	0.02	0.41	0.18, 0.95	0.04
CD4 at enrolment						
<350	1			1		
350 to 500	1.37	0.58, 3.23	0.48	1.66	0.65, 4.26	0.29
>500	1.25	0.56, 2.81	0.59	1.64	0.67, 4.02	0.28
Time on ART						
0 to 3	1			1		
3 to 6	1.12	0.51, 2.45	0.78	1.21	0.53, 2.76	0.65
6 to 9	0.77	0.31, 1.89	0.56	0.75	0.28, 1.99	0.59
9+	0.77	0.24, 2.51	0.67	1	0.28, 3.53	0.99
Eligible						
Eligible	1					
Not eligible	1.52	0.84, 2.74	0.17			
NRTI						
TDF	1			1		
ABC	2.94	0.88, 9.80	0.08	3.91	1.11, 13.75	0.03
AZT	1.04	0.57, 1.91	0.90	1.29	0.56, 2.96	0.54
NNRTI/PI						
EFV	1			1		
LPVr	2.52	0.60, 10.58	0.21	1.76	0.39, 7.99	0.46
NPV	0.86	0.47, 1.60	0.64	0.76	0.34, 1.73	0.51

The global proportional hazard test for the adjusted model was *p* = 0.85.

NRTI, nucleoside reverse transcriptase inhibitor; NNRTI, non‐nucleoside reverse transcriptase inhibitors; PI, protease inhibitor.

**Table 4:** Reasons for returning to clinical care of 96 patients

Reasons	CAGs		Comp. Outreach		Treat. Clubs		Total	
	n	%	n	%	n	%	n	%
Return to clinical care by protocol (TB, pregnancy, other medical)^a^	9	13	1	14	3	18	13	13.5
Return to clinical care by protocol violation	11	15	1	14	1	6	13	13.5
TFO to clinic without community ART	0	0	0	0	1	6	1	1
Group dissolved	19	26	0	0	0	0	19	19.8
Personal reason	17	24	3	43	0	0	21	20.8
Unknown reason	16	22	2	29	12	71	30	31.3
Total	72	100%	7	100%	17	100%	96	100%

a n = 4 develop TB, n = 5 pregnancy, n = 4 other medical.

## 4 Discussion

The implementation of community‐centred ART care models in Swaziland was feasible under routine conditions. Overall ART care retention at 12 months was 93.7% and comparable between the alternative community‐centred care models. In this cohort, a considerable proportion of patients (10.5%) returned from community ART care to clinical care, resulting in suboptimal community ART model retention. The flexibility to move between community and clinical care did not result in higher disengagement from ART, as retention in ART remained above 90% at 12 months for all three models.

Care model retention was specifically lower for the CAG model (70.4%) at 12 months likely due to a combination of operational and social patient level factors. First, 15% of patients were returned to clinical care from CAGs due to protocol eligibility violations. Second, 50% of CAG members who returned to clinical care did so because of personal reasons or because the group dissolved due to conflicts within the group members. Similar challenges were reported from another setting in Mozambique where eligibility criteria were not always respected 7, 10 and in Malawi where some CAGs collapsed due to tensions and interpersonal conflicts within the groups 4. Reportedly there was need of active involvement of health workers to manage CAGs. Although CAGs are meant to be self‐sustained, health workers needed to mediate social conflicts between CAG members and CAGs were dissolved when such conflicts could not be resolved. Although our criterion for referral back to facility‐based care was if viral load was unsuppressed (>1000 copies/mL), we noted that some patients with detectable but suppressed viral load (between 100 and 1000 copies/mL) were referred back to clinical care even though they were still eligible for these models. Although this affected retention in care model, the practice may have been beneficial to the clients given the reported predictive risk of treatment failure due to HIV drug resistance among patients whose viral load was less than 1000 copies/mL 25. Such patients received individual‐based care with possible stepped up adherence counselling to ensure undetectable viral loads are achieved.

Of concern was that health workers did not adhere to eligibility criteria for enrolment into community‐centred ART in 37.4% of cases. Patients may have pressured health workers to be included in one of the care models to benefit from less intensive clinic follow‐up. Also parents enrolled into treatment clubs reportedly pressured health workers to enrol their children in order to avoid extra clinic visits. In addition, certain patients may have been considered as stable although clinical criteria were not met. Finally, enrolment into community‐centred models may have been seen as one way to overcome barriers to adherence (e.g. for patients constrained by long waiting times at clinics for ART refill, travel expenses). The reduction in these indirect costs has been cited as the main reason for improvement for patient retention on ART 26, 27, 28. Although we did not investigate if patient groups with specific needs and barriers (e.g. adolescents) may benefit from community models, increasing interest in broadening inclusion criteria has been noticed nationally and internationally, for example Decroo et al. recommended individuals with less suppressed immunological status be included in CAGs 12. Our findings that retention in ART was not lower for ineligible patients may support a potential broadening of inclusion criteria.

The active support of the authorities was crucial for the successful implementation of community ART in this setting. For instance, allowing patients to take higher quantities of ARVs into the community raised concerns among health workers during the preparation period. Concerns were overcome through clear guidance and support provided by the National AIDS Programme to all stakeholders. Of note, not all three care models were taken up by a single facility. Choice of care model was mainly based on facility characteristics, for instance large facilities opted for treatment clubs, rural primary care clinics established CAGs and facilities already involved in comprehensive outreach activities for maternal and child welfare opted for ART integration into their routine outreach activities. Programme managers reported that combining all care models may overstretch capacity of the facilities, and health workers preferred to first gain lessons learnt to later make an informed decision when adding a second care model. In addition, it was a national requirement to start small, and to document the successes and challenges over a period of 12 months before the decision on large scale‐up. At the end of the follow‐up period, one medium‐sized primary care clinic started implementing all three care models. In addition, the Ministry of Health established guidelines on community ART 29 and started scaling up nationwide.

Currently, the fast‐track care model is also available for stable clients where clinical reviews are done every six months coupled with laboratory tests, and in between patients directly pick up their drugs from pharmacy without being seen by a clinician 29. Although not part of this analysis, this care model appears to be favoured by HCW in our setting due to the less human resource time than for establishing and maintaining the more labour‐intensive CAGs. It also appears attractive for patients due to shorter waiting time at facility level. However, this care model does not contain the peer to peer support component which allegedly is one of the advantages of the other community‐based ART models 7, 13, 14, 19, 20. A qualitative understanding of patient preference for one community ART model compared with another is warranted, but was out of the scope of this implementation and analysis.

This report has several limitations, most notably, the observational nature of the analytic design. As opposed to a randomized controlled trial, patients were enrolled into the different community ART models according to the availability of the model at their facility. We therefore cannot exclude the possibility that differences between facilities and patients also contributed to differences in retention in care model. Due to limited information on individual patients, we could not adjust for all possible differences between patients which may have biased our findings in either direction. Second, data were retrospectively abstracted from patient files and registers for analysis. While common practice, use of routinely collected data for analysis can lead to data quality issues and missing information. Lastly, we were unable to directly compare retention in community ART models to retention in facility based care. Such an analysis would need to consider the eligibility requirements for enrolling into community ART (which may also be predictors of retention in care), namely being stable on ART, high CD4 and viral suppression, among patients at the facility for a non‐biased comparison. However individual level data of patients not enrolled into community ART was not available from these facilities.

To the best of our knowledge, this is the first report describing the implementation of different community ART care models in the same setting. It is unlikely that one care model would be considered feasible in all settings, as such the models should be context specific (e.g. high vs. low patient volume facility, rural vs. urban and hard to reach communities vs. close to facilities) 21 and offer flexibility to move between the models as patient needs change over time. The three community‐centred models piloted in our setting have the potential to reduce time and financial costs incurred by frequent visits to the facilities and offer peer support as highlighted in previous studies 6, 7, 10, 11, 14, 18. The models also empower patients to take responsibility for their own health 7, decongest healthcare facilities and ultimately improve the treatment outcome of the people on ART 30, 31 Additional resources specifically for CAGs and comprehensive outreach are needed in terms of transport to provide outreach services as well as adequate support of the CAGs in the community. In addition, the CAG model may require involvement of the healthcare workers in mediating interpersonal conflicts within the groups.

## 5 Conclusion

We showed that the provision of several patient‐centred and decentralized care models was feasible in the context of constrained resources, as shown by increasing enrolment into these models over time and high retention in ART. Although access to community ART was meant to be restricted to patients stable on ART, more research is needed to assess if patients with adherence barriers and specific needs may also benefit from community‐centred models. Community‐centred ART models have the potential to alleviate the burden of high numbers of asymptomatic patients on the health system, and to reduce the travel time and costs for patients. These models may become even more relevant in the coming years as ART scale‐up continues through the WHO treat‐all approach.

## Competing interests

No competing interests declared.

## Authors’ contributions

LP, SN, GS, SM, SMK, BR, IC and BK designed and planned the research. LP, SN, GS, SM, NL, MP, SMK, BR, IC and BK performed and/or supervised the implementation. RN and LP analysed the data. LP, RN and BK wrote the first draft of the manuscript. All authors contributed to the writing and approved the final manuscript.

## Supporting information


**Table S1.** Adjusted and unadjusted Cox model estimates of all‐cause attrition (death, LTFU, disengagement from care model) in care model for eligible patients only (n = 575).
**Table S2.** Unadjusted and adjusted Cox model estimates of all‐cause ART attrition (death, LTFU) for eligible patients only (n = 575).
**Table S3.** Outcomes by model type for eligible patients only (n = 575; chi square test *p* = 0.15).
