# Supplementary material for: Retention on ART and predictors of disengagement from care in several alternative community‐centred ART refill models in rural Swaziland
Source: J Int AIDS Soc. 2018 Sep 18;21(9):e25183. doi: 10.1002/jia2.25183 (PMC6141897; doi:10.1002/jia2.25183)
Supplement: Supplementary file 1 — Table S1. Adjusted and unadjusted Cox model estimates of all‐cause attrition (death, LTFU, disengagement from care model) in care model for eligible patients only (n = 575). Table S2. Unadjusted and adjusted Cox model estimates of all‐cause ART attrition (death, LTFU) for eligible patients only (n = 575). Table S3. Outcomes by model type for eligible patients only (n = 575; chi square test p = 0.15). [file JIA2-21-e25183-s001.docx]

**Appendix**

**Table S1.** Adjusted and unadjusted Cox model estimates of all cause attrition (death, LTFU, disengagement from care model) in care model for eligible patients only, n=575.

|  | | **Unadjusted estimates** | | | **Adjusted model n=574** | | |
| --- | --- | --- | --- | --- | --- | --- | --- |
|  |  | **HR** | **95% CI** | **p** | **HR** | **95% CI** | **p** |
| **Model** | CAG | 3.82 | 2.18,6.66 | p<0.001 | 4.29 | 2.38,7.71 | p<0.001 |
|  | Outreach | 1.75 | 0.76,4.04 | 0.19 | 2.00 | 0.84,4.75 | 0.12 |
|  | Treat. Clubs | 1 |  |  | 1 |  |  |
| **Gender** | Male | 1 |  |  | 1 |  |  |
|  | Female | 0.76 | 0.45,1.28 | 0.31 | 0.88 | 0.51,1.51 | 0.65 |
| **Age group (years)** | <24 | 2.06 | 0.64,6.61 | 0.23 | 1.2 | 0.36,3.96 | 0.76 |
|  | 25-49 | 1 |  |  | 1 |  |  |
|  | 50+ | 0.79 | 0.47,1.33 | 0.37 | 0.68 | 0.40,1.16 | 0.16 |
| **CD4 at enrolment** | 350-500 | 1 |  |  | 1 |  |  |
|  | >500 | 1.06 | 0.66,1.71 | 0.81 | 1.09 | 0.67,1.77 | 0.74 |
| **Time on ART** | 0-3 | 1 |  |  | 1 |  |  |
|  | 3-6 | 1.26 | 0.65,2.42 | 0.50 | 1.52 | 0.77,3.00 | 0.23 |
|  | 6-9 | 1.05 | 0.52,2.12 | 0.89 | 1.32 | 0.60,2.89 | 0.48 |
|  | 9+ | 0.33 | 0.09,1.16 | 0.08 | 0.42 | 0.12,1.54 | 0.19 |
| **NRTI** | TDF | 1 |  |  | 1 |  |  |
|  | ABC | 1.71 | 0.53,5.54 | 0.37 | 1.89 | 0.56,6.35 | 0.30 |
|  | AZT | 0.92 | 0.57,1.49 | 0.75 | 0.9 | 0.47,1.74 | 0.76 |
| **NNRTI/ PI** | EFV | 1 |  |  | 1 |  |  |
|  | LPVr | 1.4 | 0.34,5.76 | 0.64 | 1.83 | 0.42,7.99 | 0.42 |
|  | NPV | 0.73 | 0.45,1.19 | 0.21 | 0.85 | 0.45,1.59 | 0.60 |

**Table S2.** Unadjusted and adjusted Cox model estimates of all cause ART attrition (death, LTFU) for eligible patients only, n=575.

|  | | **Unadjusted estimates** | | | **Adjusted model n=574** | | |
| --- | --- | --- | --- | --- | --- | --- | --- |
|  |  | **HR** | **95% CI** | **p** | **HR** | **95% CI** | **p** |
| **Model** | CAG | 1.07 | 0.55,2.09 | 0.84 | 1.1 | 0.44,2.77 | 0.84 |
|  | Outreach | 1.57 | 0.70,3.53 | 0.27 | 2.11 | 0.69,6.45 | 0.19 |
|  | Treat. Clubs | 1 |  |  | 1 |  |  |
| **Gender** | Male | 1 |  |  | 1 |  |  |
|  | Female | 0.63 | 0.27,1.45 | 0.28 | 0.59 | 0.24,1.41 | 0.23 |
| **Age-group years** | <24 | 3.54 | 0.83,15.15 | 0.09 | 1 |  |  |
|  | 25-49 | 1 |  |  | 1.3 | 0.56,3.01 | 0.54 |
|  | 50+ | 0.39 | 0.13,1.14 | 0.08 | 3.99 | 0.85,18.73 | 0.08 |
| **CD4 at enrolment** | 350-500 | 1 |  |  | 1 |  |  |
|  | >500 | 1.18 | 0.53,2.65 | 0.68 | 0.38 | 0.13,1.13 | 0.08 |
| **Time on ART** | 0-3 | 1 |  |  | 1 |  |  |
|  | 3-6 | 1.37 | 0.45,4.21 | 0.58 | 1.56 | 0.49,4.98 | 0.45 |
|  | 6-9 | 1.15 | 0.35,3.83 | 0.82 | 1.47 | 0.38,5.70 | 0.58 |
|  | 9+ | 0.34 | 0.04,3.02 | 0.33 | 0.52 | 0.05,4.97 | 0.57 |
| **NRTI** | TDF | 1 |  |  | 1 |  |  |
|  | ABC | 3 | 0.68,13.19 | 0.15 | 3.25 | 0.67,15.78 | 0.14 |
|  | AZT | 0.82 | 0.37,1.85 | 0.64 | 1.03 | 0.35,3.03 | 0.96 |
| **NNRTI/ PI** | EFV | 1 |  |  | 1 |  |  |
|  | LPVr | 1.68 | 0.22,12.62 | 0.61 | 1.21 | 0.15,9.85 | 0.86 |
|  | NPV | 0.59 | 0.25,1.37 | 0.22 | 0.58 | 0.21,1.59 | 0.29 |
|  | | | | | | | |

**Table S3.** Outcomes by model type for eligible patients only, n= 575 (chi square test p=0.15).

|  | **CAG** | | **Comprehensive Outreach** | | **Treatment Club** | |
| --- | --- | --- | --- | --- | --- | --- |
| **Outcome** | **N** | **%** | **N** | **%** | **N** | **%** |
| **RIP** | 1 | 0.34% | 1 | 1.69% | 1 | 0.46% |
| **LTFU** | 5 | 1.68% | 3 | 5.08% | 8 | 3.65% |
| **TFO** | 2 | 0.67% | 1 | 1.69% | 1 | 0.46% |
| **Return to clinical** | 40 | 13.47% | 4 | 6.78% | 9 | 4.11% |
| **Retained in care** | 249 | 83.84% | 50 | 84.75% | 200 | 91.32% |
